# Operative and Oncological Outcomes Comparing Sentinel Node Mapping and Systematic Lymphadenectomy in Endometrial Cancer Staging: Meta-Analysis With Trial Sequential Analysis

**DOI:** 10.3389/fonc.2020.580128

**Published:** 2021-01-13

**Authors:** Yu Gu, Hongyan Cheng, Liju Zong, Yujia Kong, Yang Xiang

**Affiliations:** ^1^Department of Obstetrics and Gynecology, Peking Union Medical College Hospital, Chinese Academy of Medical Sciences and Peking Union Medical College, Beijing, China; ^2^Department of Pathology, Peking Union Medical College Hospital, Chinese Academy of Medical Sciences and Peking Union Medical College, Beijing, China

**Keywords:** endometrial cancer, sentinel node mapping, lymphadenectomy, operation, lymph node assessment, oncological outcome

## Abstract

**Objective:**

To evaluate the utility of sentinel lymph node mapping (SLN) in endometrial cancer (EC) patients in comparison with lymphadenectomy (LND).

**Methods:**

Comprehensive search was performed in MEDLINE, EMBASE, CENTRAL, OVID, Web of science databases, and three clinical trials registration websites, from the database inception to September 2020. The primary outcomes covered operative outcomes, nodal assessment, and oncological outcomes. Software Revman 5.3 was used. Trial sequential analysis (TSA) and Grading of Recommendations Assessment, Development, and Evaluation (GRADE) were performed.

**Results:**

Overall, 5,820 EC patients from 15 studies were pooled in the meta-analysis: SLN group (N = 2,152, 37.0%), LND group (N = 3,668, 63.0%). In meta-analysis of blood loss, SLN offered advantage over LND in reducing operation bleeding (I^2^ = 74%, P<0.01). Z-curve of blood loss crossed trial sequential monitoring boundaries though did not reach TSA sample size. There was no difference between SLN and LND in intra-operative complications (I^2^ = 7%, P = 0.12). SLN was superior to LND in detecting positive pelvic nodes (P-LN) (I^2^ = 36%, P<0.001), even in high risk patients (I^2^ = 36%, P = 0.001). While no difference was observed in detection of positive para-aortic nodes (PA-LN) (I^2^ = 47%, P = 0.76), even in high risk patients (I^2^ = 62%, P = 0.34). Analysis showed no difference between two groups in the number of resected pelvic nodes (I^2^ = 99%, P = 0.26). SLN was not associated with a statistically significant overall survival (I^2^ = 79%, P = 0.94). There was no difference in progression-free survival between SLN and LND (I^2^ = 52%, P = 0.31). No difference was observed in recurrence. Based on the GRADE assessment, we considered the quality of current evidence to be moderate for P-LN biopsy, low for items like blood loss, PA-LN positive.

**Conclusion:**

The present meta-analysis underlines that SLN is capable of reducing blood loss during operation in regardless of surgical approach with firm evidence from TSA. SLN mapping is more targeted for less node dissection and more detection of positive lymph nodes even in high risk patients with conclusive evidence from TSA. Utility of SLN yields no survival detriment in EC patients.

## Highlights

- SLN is capable of reducing blood loss during operation in regardless of surgical approach with firm evidence from TSA.
- SLN mapping is more targeted for less node dissection and more detection of positive lymph nodes even in high risk patients with conclusive evidence from TSA.
- Utility of SLN yields no survival detriment in EC patients.

## Introduction

Endometrial cancer (EC) is the most common gynecological malignancy in developed countries, and an estimated 65,620 new cases in United States in 2020 (1). The disease incidence has been climbing by 1.5 times over the last 10 years, and the death cases have increased by 58.4% according to latest statistics (1, 2). Though 5-year overall survival (OS) has reached at 80%, it has not made any progress since 1985, estimated in the US Surveillance, Epidemiology, and End Results (SEER) database (3).

Surgical staging is the step of final diagnosis and first treatment in most EC patients, and the standard operation includes hysterectomy, bilateral salpingo-oophorectomy, and lymph node assessment, allowing prognostic stratification and potentially benefited patients identification (3).

Lymph node status is a definite prognostic factor, albeit clinical trials showed no survival benefit in patients with nodal examination *versus* those not (4, 5). Ongoing controversy remains the extent of nodal dissection to tailor post-operation therapy. Traditional lymph node assessment contains systematic pelvic ± para-aortic lymphadenectomy (LND), and given low lymph nodal involvement rate, LND is prone to cause overtreatment and thus more surgery-related complications like lymphedema (6).

Sentinel lymph node mapping (SLN) has emerged as a reliable alternative in EC nodal assessment. Accumulating studies have demonstrated SLN was equal to LND in low- and high-risk EC patients and oncological outcomes were similar in both SLN and LND groups (7, 8). It has been recommended in low- and high-risk EC patients for surgical staging procedures in 2020 National Comprehensive Cancer Network (NCCN) guidelines (9). The superiority of SLN lies in pathological ultra-staging to avoid overtreatment and undertreatment.

A previous meta-analysis indicated SLN was superior to LND in nodal assessment (10), but given its limited data, further discussion about operative and oncological outcomes is still needed. The aim of this meta-analysis was to systematically review current evidence in comparison of two nodal assessment technologies, SLN and LND, in EC patients. The main outcomes contain surgery-related outcomes, nodal assessment, and oncological outcomes.

## Materials and Methods

This analysis has been registered in the International Prospective Register of Systematic Reviews (PROSPERO, https://www.crd.york.ac.uk/prospero, ID: CRD42020175099). And this meta-analysis was completed by the Preferred Reporting Items for Systematic Reviews and Meta-Analyses (PRISMA) guidelines (11).

### Search Strategy

Comprehensive search was performed in MEDLINE, EMBASE, CENTRAL, OVID, Web of science databases, from the database inception to September 2020. The key words included “endometrial cancer,” “sentinel node,” and “lymphadenectomy”. And three clinical trials registration websites, the Clinical trials.gov (www.clinicaltrials.com), WHO trial website (https://apps.who.int/trialsearch), and the Controlled Trials meta Register (www.controlled-trials.com), were searched as well. Details of search strategy is shown in **Supplement Files S1**.

### Inclusion and Exclusion Criteria

Two independent reviewers (YG and HC) conducted selection of studies based on a protocol defined priorly. Studies were included if they met the following criteria: 1) patients diagnosed with endometrial cancer; 2) clinical trials concerning the comparison of sentinel node mapping and lymphadenectomy; 3) reported operative outcomes like operative time, blood loss, operative complications; lymph nodes assessment like the number of positive pelvic lymph nodes; oncological outcomes like overall survival and recurrence, but not limited to these above. The exclusion criteria as: 1) <10 patients; 2) review, case report, comment, and other types without original data; 3) full text could not be obtained; 4) written other than in English. At first screening, titles and abstracts of articles were assessed according to inclusion and exclusion criteria. Then full texts were read to identify eligibility. Consensus was made by discussion when disagreement occurring.

### Data Extraction

Data were extracted using a modified form based on the Cochrane reviews handbook. The following information was collected: author, year of publication, study design, patients’ characteristics, surgical approach, SLN technique, operative outcomes, nodal assessment and oncological outcomes, and so forth. Two reviewers (YG and LZ) conducted date extraction independently, and inconformity was resolved by discussion.

### Quality Assessment

The Newcastle-Ottawa scale for cohort study was used for article quality assessment. This scale is comprised of three parts (selection, comparability, and outcome). With a maximum of nine stars, articles reaching six stars were included finally. Two reviewers (YG and YK) assessed articles independently, and consensus was reached by discussion in the event of disparity (**Supplement Files S2**).

### Statistical Analysis

Software Revman 5.3 was used to pool data and generate forest plots. Mantel-Haenszel method was used in dichotomous data and the odds ratio (OR) was calculated. And for continuous data inverse variance and mean difference (MD) were applied. Random-effect model was used in analysis. Heterogeneity of included studies was assessed by I^2^ and I^2^>50% was defined as high heterogeneity. Subgroup analysis by SLN procedure or patients risk stratification was introduced when meeting high heterogeneity. When necessary, data, like operative time or blood loss, in form of (median, range) were transformed into (mean, standard difference) according to recommended methods (12). Hazard ratio (HR) and 95% confidence interval (CI) of overall survival (OS) and progression-free survival (PFS) were extracted from Kaplan-Meier curve using Engauge Digitizer software (10.7) and recommended methods (13). And for data failing to conducting meta-analysis, a narrative systemic review was performed. Trial sequential analysis (TSA) was performed by TSA software (version 0.9β) and we calculated sample size adjusted for this meta-analysis to testify whether the evidence is confirmed and conclusive. Pooled analysis was graded by the Grading of Recommendations, Assessment, Development and Evaluation (GRADE) approach, and the certainty of evidence was assessed as high, moderate, low, or very low, using GRADE pro website (https://gdt.gradepro.org).

## Results

**Figure 1:**
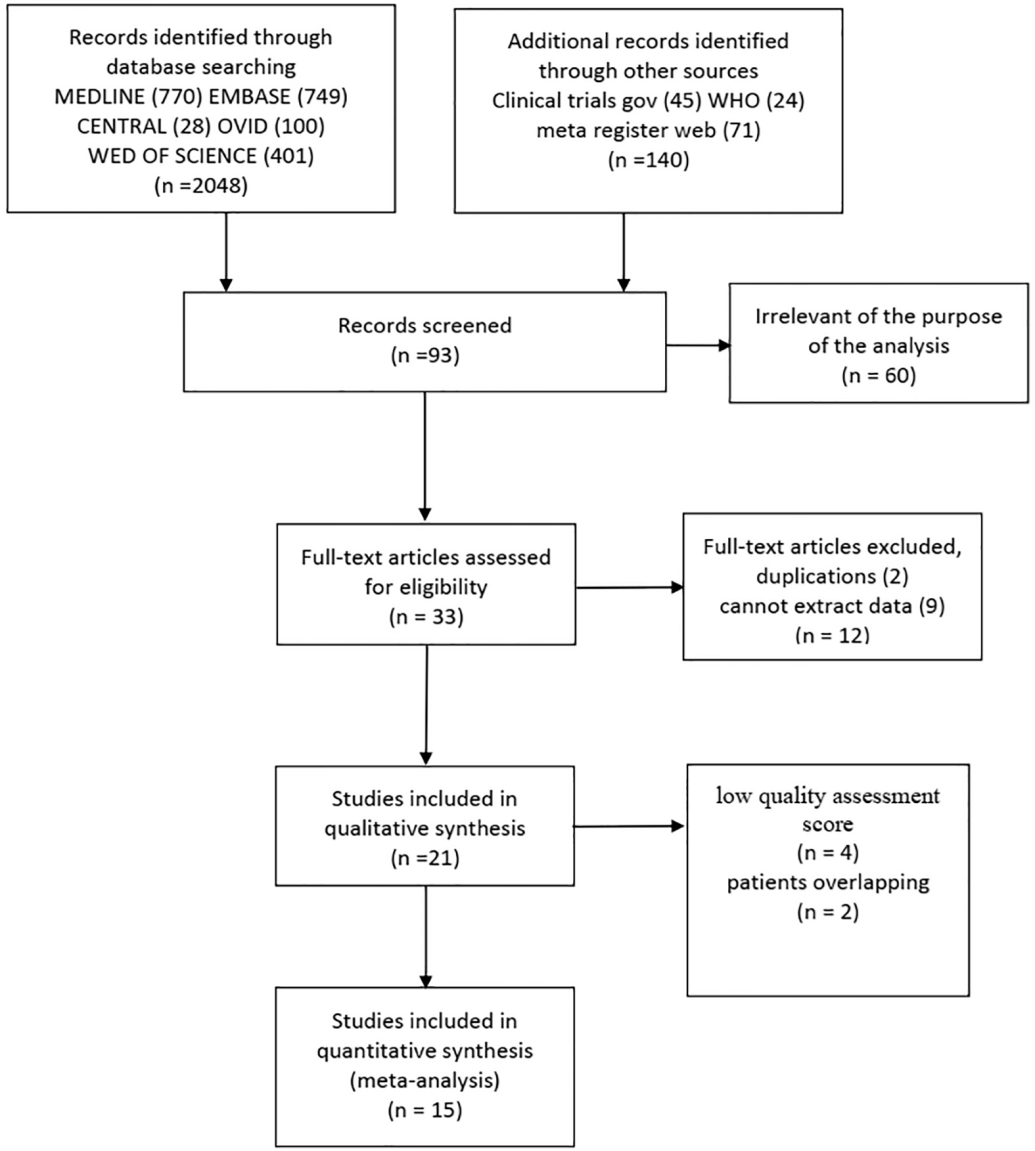
Selection of studies for inclusion in the systematic review.

A total of 2,048 articles were screened through the search strategy, and 21 articles were included after full text reading. Four were excluded for low quality assessment score (<6) (14–17), two were excluded for patients overlapping (18, 19), thus leaving 15 articles eligible for final analysis (7, 20–33) (**Figure 1**). Characteristics of the 15 studies are summarized in **Supplement Table S1**. Overall, 5,820 EC patients were pooled in the meta-analysis: SLN group (N = 2,152, 37.0%), LND group (N = 3,668, 63.0%), respectively.

### Operative Outcomes

**Figure 2:**
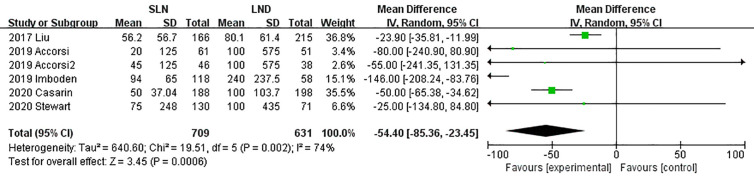
Meta-analysis of blood loss.

**Figure 3:**
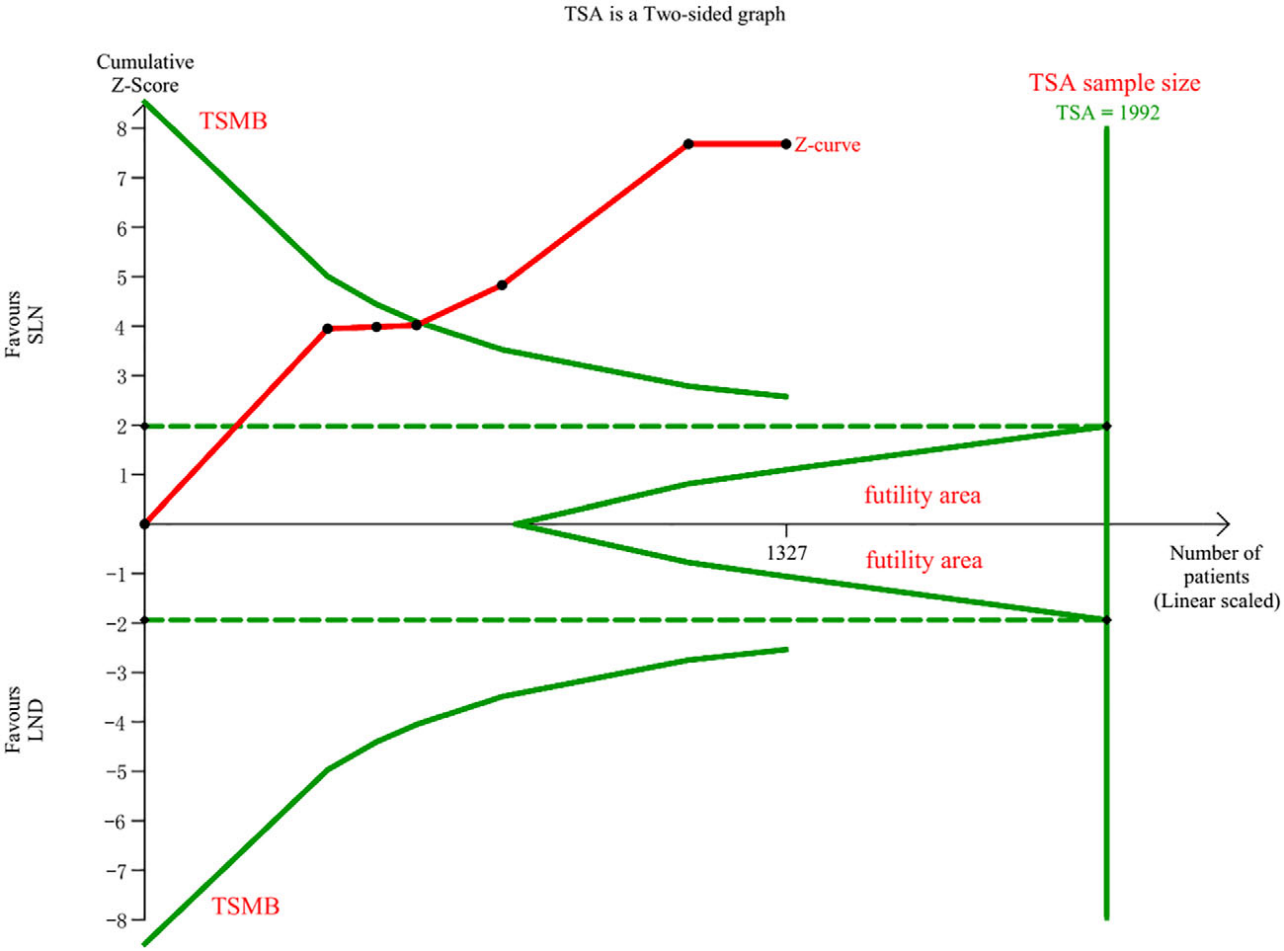
TSA of blood loss, α = 0.05, β = 0.8, two-sided test.

**Figure 4:**
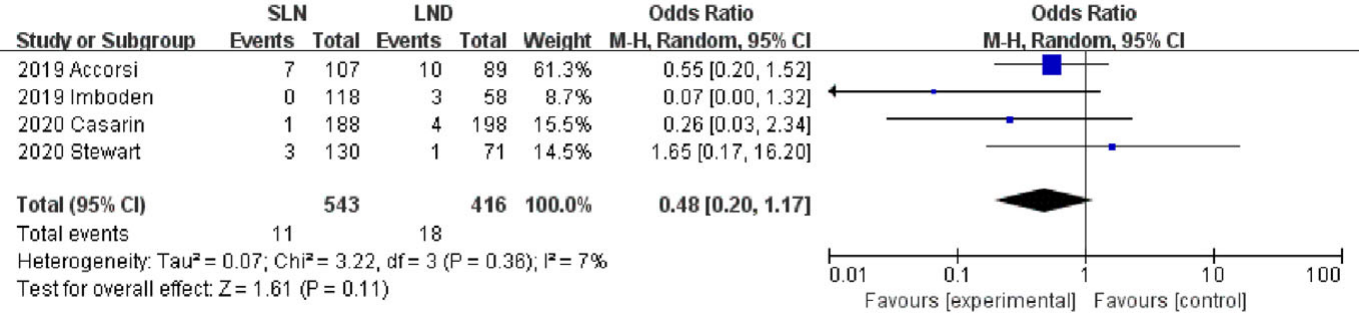
Meta-analysis of intra-operative complications.

Data regarding operation related outcomes were available in seven studies (**Supplement Table S2**). In meta-analysis of blood loss, SLN offered advantage over LND in reducing operation bleeding; the MD was −54.40, 95% CI −85.36~−23.45 (I^2^ = 74%, P<0.001; **Figure 2**). Z-curve of blood loss crossed trial sequential monitoring boundaries (TSMB) though did not reach TSA sample size, and indicating the result was true-positive (**Figure 3**). When intra-operative complications were measured, there was no difference between SLN and LND (I^2^ = 7%, P=0.11, **Figure 4**).

When operative time was pooled, subgroup analysis failed to identify high heterogeneity (I^2^ = 99%, P<0.01, **Supplement Figure 1A**), but a tendency of shorter operative time in SLN group was shown in **Supplement Table S2**. And TSA of operative time showed Z-curve crossed TSMB and highly surpassed TSA sample size (**Supplement Figure 1B**). Post-operative complications were assessed by Accordion Severity Grading System, Clavien-Dindo scale and MSKCC’s Surgical Secondary Events Grading System. It seemed that SLN group had lower post-operative complications but more data are needed to conduct further analysis. When considering conversion rate, re-admission, re-operation, length of stay and frozen utility, potential advantage of SLN could be seen in shortening length of stay and frozen utility (**Supplement Figures 1C, D**). And TSA of length of stay showed inconclusive result for insufficient sample size (**Supplement Figure 1E**). Additionally, TSA of post-operative complications, conversate rate, re-admission, re-operation, and frozen utility were available for low sample size.

### Lymph Node Assessment

**Figure 5:**
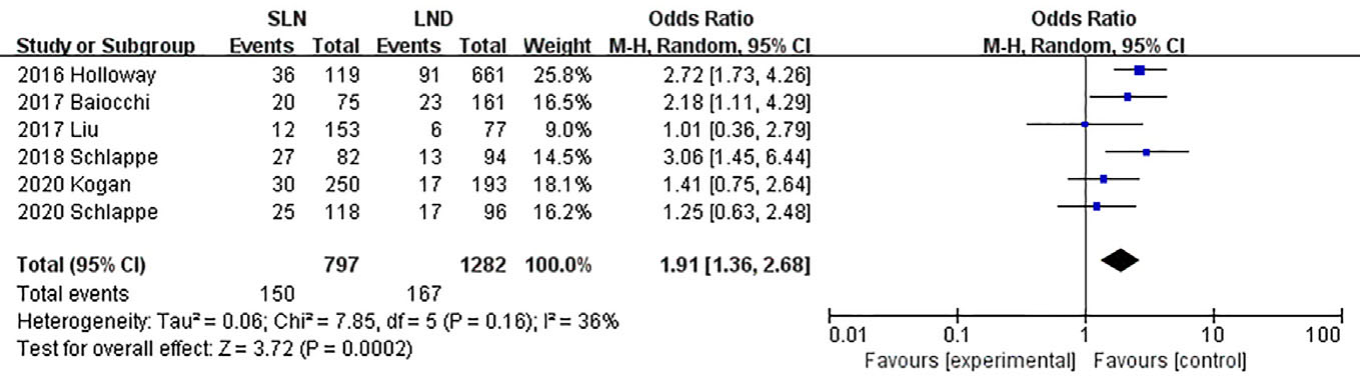
Meta-analysis of pelvic lymph nodes positive.

**Figure 6:**
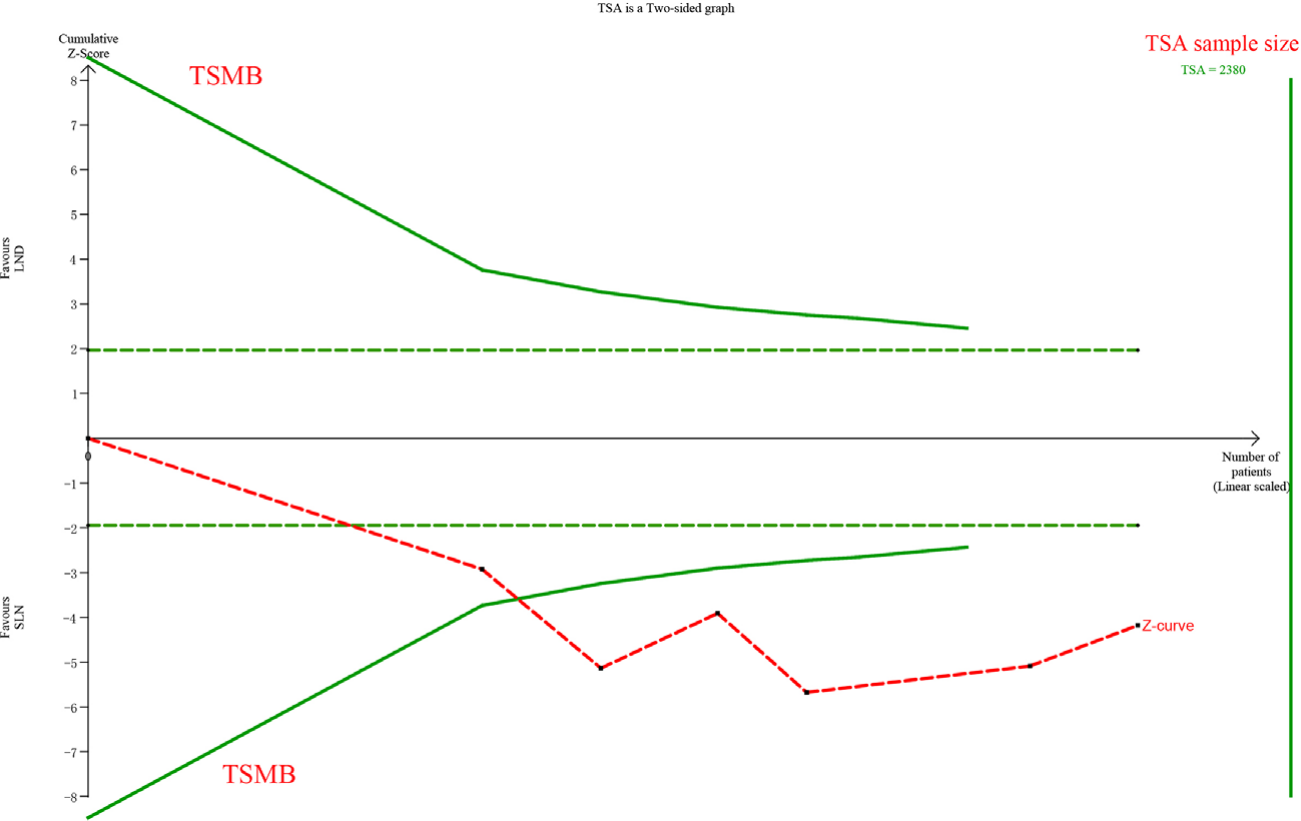
TSA of pelvic lymph nodes, α = 0.05, β = 0.8, relative risk reduction = −73.8%, incidence in control group = 16.4%, two-sided test.

**Figure 7:**
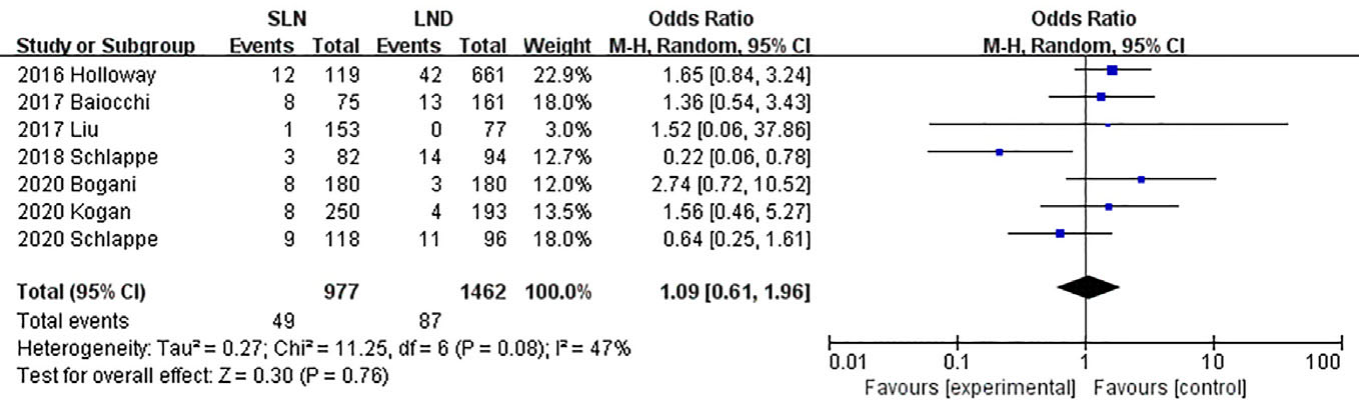
Meta-analysis of para-aortic lymph nodes positive.

**Figure 8:**
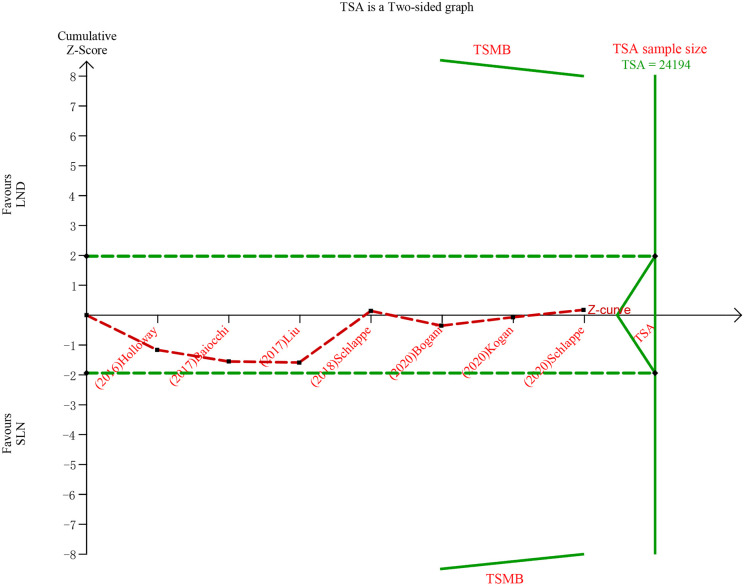
TSA of para-aortic lymph nodes, α = 0.05, β = 0.8, relative risk reduction = 15.4%, incidence in control group = 9.2%, two-sided test.

The meta-analysis of nodal assessment was based on 10 trials (**Supplement Table S3**). SLN was superior to LND in detecting positive pelvic lymph nodes (I^2^ = 36%, P<0.001, **Figure 5**). The Z-curve crossed TSMB and did not reach TSA sample size, and indicating the result was conclusive (**Figure 6**). While no difference was observed in detection of positive para-aortic nodes between two groups (I^2^ = 47%, P = 0.76, **Figure 7**); and Z-curve did not cross TSMB and did not reach TSA sample size, and indicating the result was under discussion (**Figure 8**). In high risk patients, SLN had a higher pelvic nodes detection rate (OR 2.00, 95% CI 1.21~3.32, I^2^ = 36%, P = 0.007, **Supplement Figure 2A**) and showed no difference in para-aortic nodes detection (OR 0.62, 95% CI 0.24~1.64, I^2^ = 62%, P = 0.34, **Supplement Figure 2B**).

**Figure 9:**
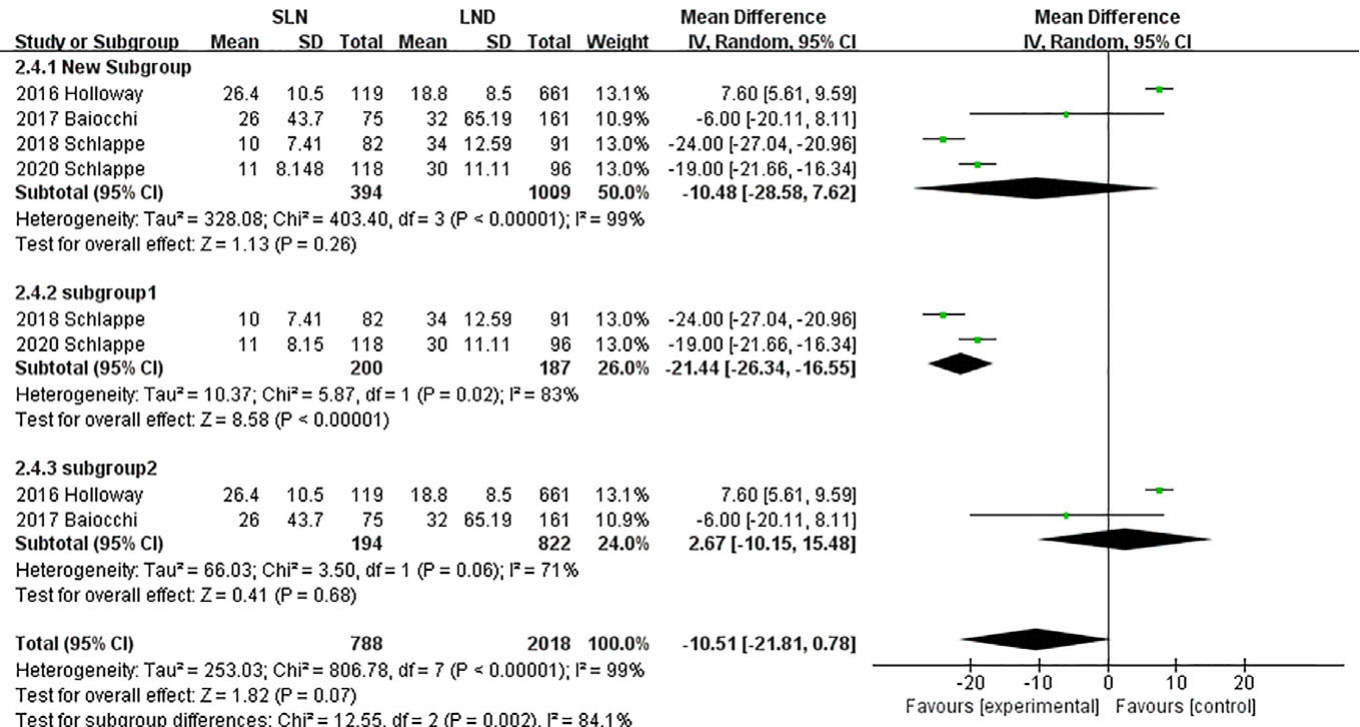
Meta-analysis of pelvic lymph nodes removed.

**Figure 10:**
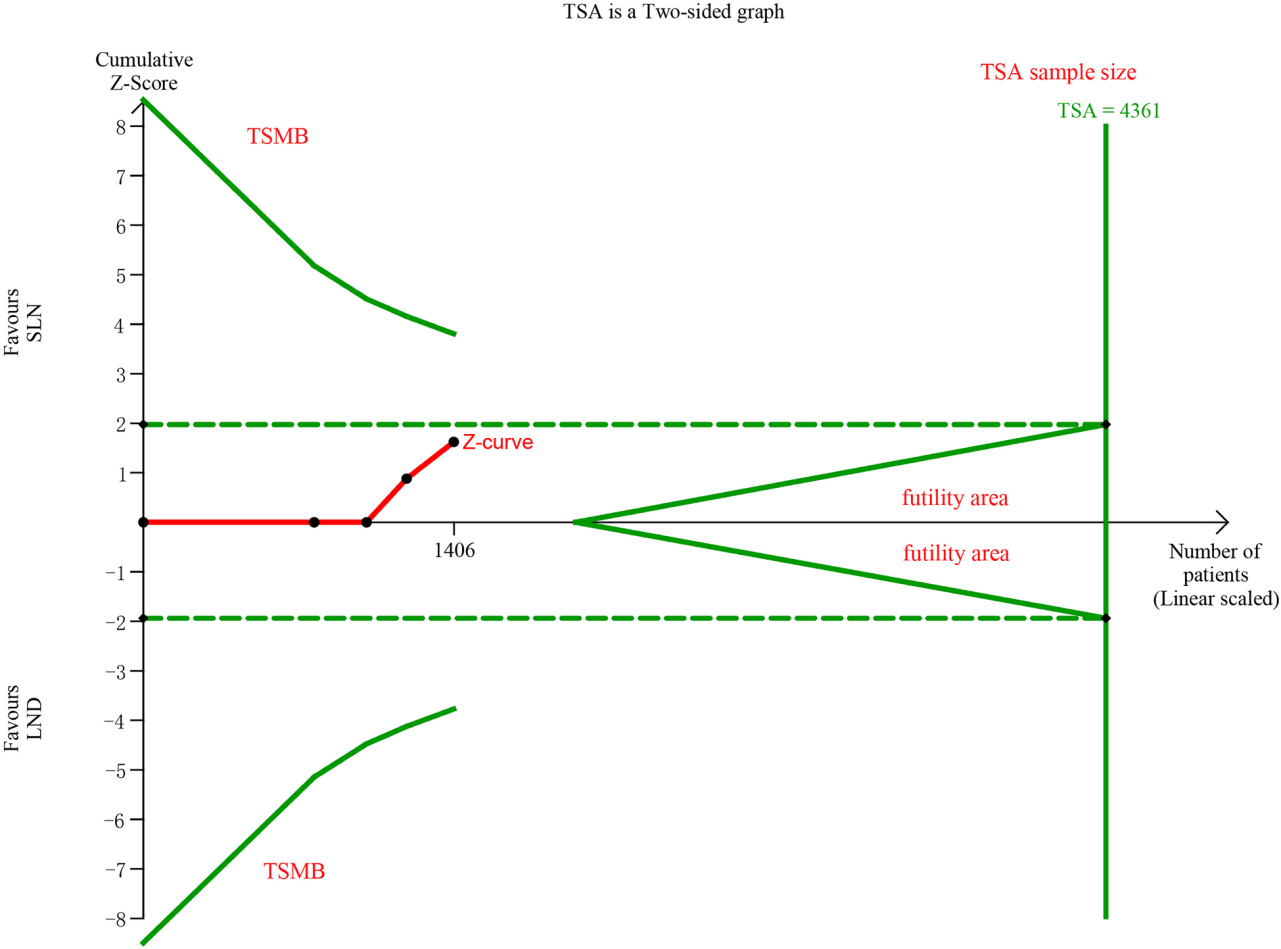
TSA of pelvic lymph nodes removed, α = 0.05, β = 0.8, two-sided test.

**Figure 11:**
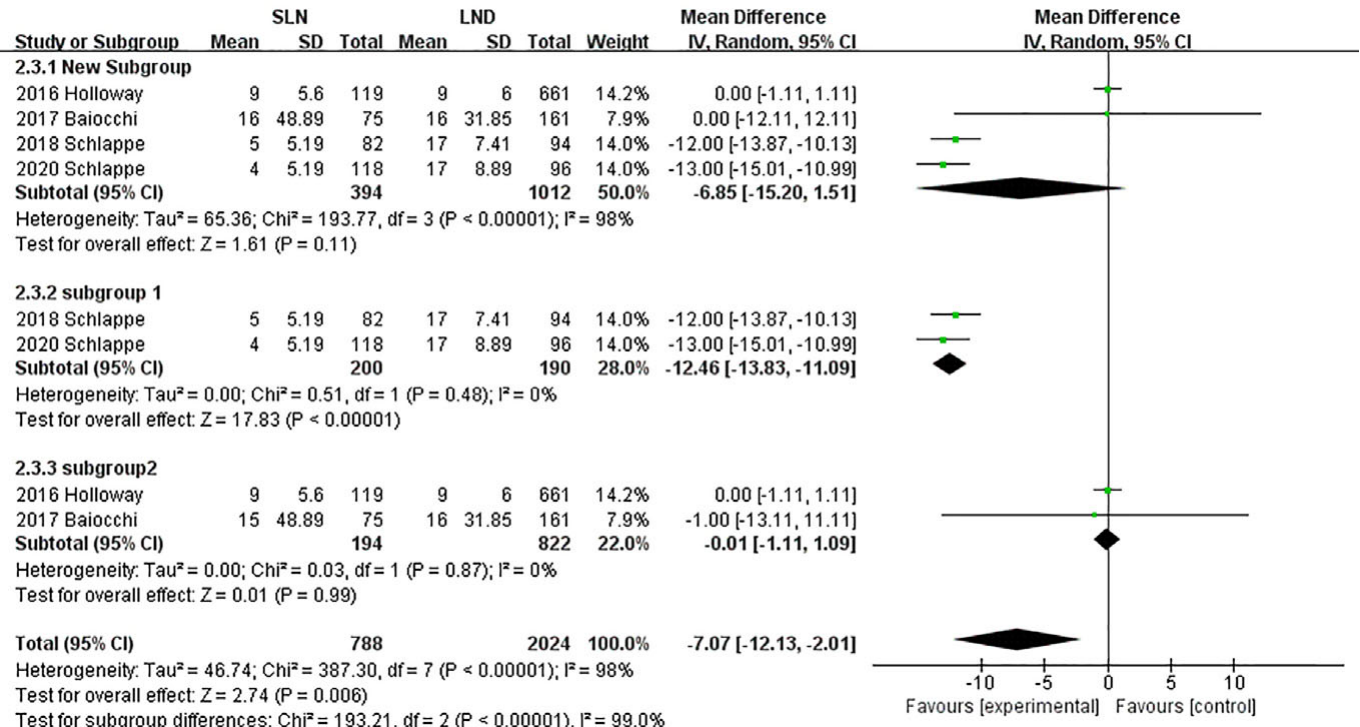
Meta-analysis of para-aortic lymph nodes removed.

**Figure 12:**
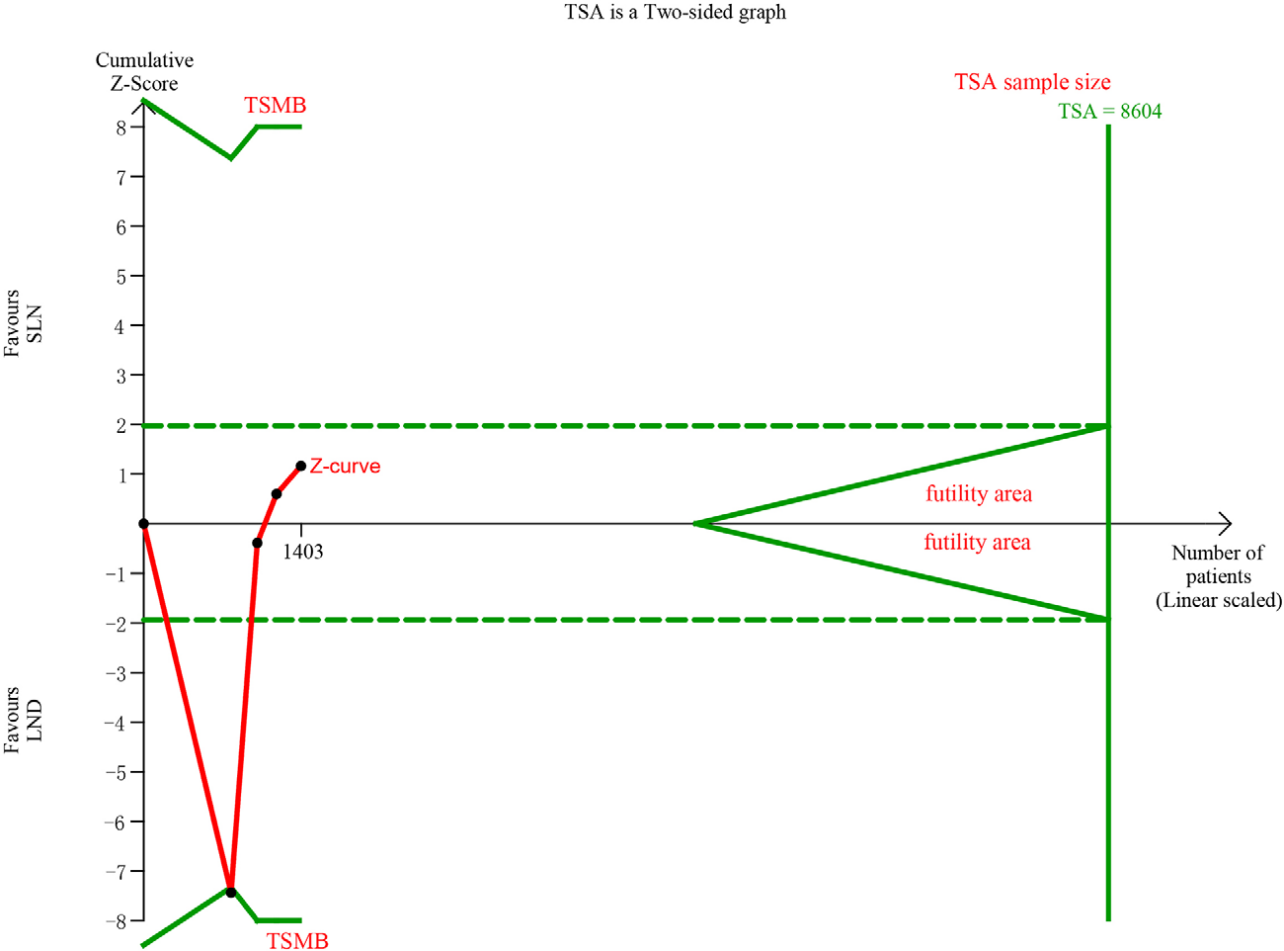
TSA of para-aortic lymph nodes removed, α = 0.05, β = 0.8, two-sided test.

In pooling data of resected pelvic nodes, analysis showed no difference between two groups (I^2^ = 99%, P = 0.26). Considering two SLN algorithm (subgroup1 SLN ± P-LND ± PA-LND; subgroup2 SLN+P-LND ± PA-LND) existed, subgroup analysis by SLN procedure was conducted and indicated that SLN procedure (SLN ± P-LND ± PA-LND) removed less pelvic nodes than LND(I^2^ = 83%, P<0.01, **Figure 9**), and Z-curve did not cross TSMB and did not reach TSA sample size, and indicating more studies were needed (**Figure 10**). The same subgroup analysis was undergone in pooling data of resected para-aortic nodes as well, and similar result was observed that SLN procedure removed less para-aortic nodes (I^2^ = 0%, P<0.001, **Figure 11**), and Z-curve did not cross TSMB and did not reach TSA sample size, and indicating more studies were needed (**Figure 12**).

### Oncological Outcomes

**Supplement Table S4** reports data concerning disease characteristics. SLN was not associated with a statistically significant OS (I^2^ = 79%, P = 0.81, **Supplement Figure 3A**). There was no difference in PFS between SLN and LND groups (I^2^ = 52%, P = 0.31, **Supplement Figure 3B**). No difference was observed in overall recurrence (all sites, I^2^ = 75%, P = 0.41, **Supplement Figure 3C**), and Z-curve did not cross TSMB and did not reach TSA sample size, and indicating more studies were needed (**Supplement Figure 3D**).

In terms of recurrence pattern, analysis of nodal recurrence, locoregional recurrence, and multifocal recurrence showed no differences between SLN and LND (P>0.05); TSA showed further studies were needed (**Supplement Figures 4A–E**). Data on death of disease was available for two trials, and meta-analysis showed no difference between two groups (P>0.05, **Supplement Figure 5**).

### GRADE Assessment

Based on the GRADE assessment, we considered the quality of current evidence to be moderate for P-LN biopsy, low for items like blood loss, PA-LN positive. We postulated that because of basis on cohort studies, the grading hardly reached higher (**Table 1**).

**Table 1:** GRADE evidence profile: Quality assessment.

Certainty assessment							Number of patients		Effect		Certainty	Importance
Number of studies	Study design	Risk of bias	Inconsistency	Indirectness	Imprecision	Other considerations	SLN(n)	LND(n)	Relative(95% CI)	Absolute(95% CI)		
**Operation time**												
8	Observational studies	Not serious	Serious^a^	Not serious	Not serious	None	959	2,243	–	MD 75.37 lower (106.36 lower to 44.38 lower)	⨁ 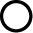 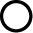 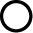 VERY LOW	CRITICAL
**Blood loss**												
6	Observational studies	Not serious	Not serious	Not serious	Not serious	None	709	631	–	MD 54.4 lower (85.36 lower to 23.45 lower)	⨁⨁ 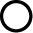 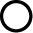 LOW	CRITICAL
**P-LN positive**												
6	Observational studies	Not serious	Not serious	Not serious	Not serious	strong association^b^	150/797 (18.8%)^c^	167/1,282 (13.0%)	OR 1.91 (1.36 to 2.68)	92 more per 1,000 (from 39 more to 156 more)	⨁⨁⨁ 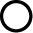 MODERATE	CRITICAL
**PA-LN positive**												
7	Observational studies	Not serious	Not serious	Not serious	Not serious	None	49/977 (5.0%)	87/1,462 (6.0%)	OR 1.09 (0.61to 1.96)	5 fewer per 1,000 (from 21 fewer to 51 more)	⨁⨁ 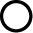 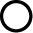 LOW	CRITICAL

GRADE, Grading of recommendation assessment, development, and evaluation; n, number of patients; CI, Confidence interval; MD, Mean difference; RR, Risk ratio; OR, Odds ratio; HR, Hazard Ratio; ^a^Heterogeneity = 99%; ^b^RR>2; ^c^[n/total n (%)].

## Discussion

The analysis reviewed present evidence in comparison of SLN and LND in EC patients, and the main findings covered operation-related outcomes, nodal assessment, and oncological outcomes. To our knowledge, this is the first meta-analysis discussing surgery-related outcomes, and found SLN was capable to reduce blood loss with firm evidence from TSA. The pooled date validates that SLN allows an accurate detection of positive lymph nodes in circumstance of less node removal, especially with conclusive evidence from TSA of P-LN positive patients. Additionally, no difference is observed in OS, PFS, and recurrence between two procedures, and TSA of recurrence showed further investigation are needed.

SLN has been gaining popularity in gynecological cancer staging over the past decades. Initial exploration of SLN by gynecological oncologist started at vulvar cancer (34) and subsequent studies validated its feasibility (35). It has experienced two stages of SLN employment in EC staging, and in the first stage multiple researches were focusing on the feasibility and reliability of SLN. Abu-Rustum et al. (36) identified a 100% sensitivity and low false-negative rate in grade 1 EC patients, with the methods of SLN procedure followed by systematic pelvic and para-aortic LND. After accumulating studies indicating the accuracy of SLN in EC staging, SLN has been evolved as a more targeted alternative for nodal assessment (37, 38). In 2014, SLN was firstly recommended by NCCN guidelines to stage I patients (39). A meta-analysis reported a>80% overall detection rate of SLN (40). A consensus from the Society of Gynecological Oncology (SGO) in 2017, approved the execution of SLN in low-risk patients (41). Till 2018, NCCN guidelines began to support SLN application in all EC patients including those with high risk (42). At present the discussion about SLN *vs.* LND is still going on (43). A recent meta-analysis highlighted the safety and effectiveness of SLN (10), but given its limitation we conducted this analysis with latest evidence, bigger sample size and border outcomes measures.

SLN is introduced into EC staging with the aim of reducing LND-related morbidity and gaining prognostic factors of lymph node status (41). SLN techniques has been evolving during gynecologic oncology application. Three different tracers, patent blue, technetium 99, and indocyanine green (ICG), are the mainstay of SLN mapping (41). Considering the unreliability and radiation, the SGO recommended ICG dye with infrared imaging to EC patients for its high success and technical ease (41). The optimal site for tracer injection has been investigated in precious studies (44–46), out of common sites like myometrium, cervix injection is regarded as the most effective way to trace SLN (41).

Recent studies focusing on operation-related outcomes indicated that SLN procedure could reduce operative time of minimal invasive surgery and laparotomy (23, 25, 26, 28, 30, 33). Stewart et al. (33) observed significant decrease in operative time (210 *vs* 170 min, P = 0.007) taking account of the surgery approach. And Valerio G. et al. (47) discussed robotic surgery in elderly gynecological cancer patients and demonstrated that minimally invasive could considered for older patients (even over 75 years old); and this illustrated SLN procedure during minimal invasive surgery could benefit patients more, especially these over 75 years old. The meta-analysis indicated that blood loss was significantly lower in SLN group. In terms of complications, more evidence is needed for supporting intra- and post-operation complications declining. Accordion Severity Grading System and Clavien-Dindo scale are used to assess post- operation complications; two studies by Accordion Severity Grading System (30, 33) and two by Clavien-Dindo scale (26, 28) indicated SLN group occurred less post-operation complications. A retrospective study reported lower-limb lymphedema could only been seen in LND group (25). Leitao et al. (48) concluded that SLN was independently related to lower self-reported lower-extremity lymphedema rate than LND.

The meta-analysis indicates that SLN is more targeted for less node dissection and more detection of positive lymph nodes. The FIRES trial, enrolling stage I patients, yielded a high sensitivity of 97% and a negative predictive value of 99.6% by SLN mapping, and prevented more people from the morbidity of LND (38). Accumulating data indicated SLN was significantly associated with accurate detection of pelvic lymph nodes and was non-inferior to LND in para-aortic nodes assessment in high risk EC patients (7, 18, 21–23, 32, 41). The pathologic ultra-staging technique adopted by SLN mapping, defines positive lymph node as macro-metastasis (≥2 mm), micro-metastasis (≥0.2 mm), and isolated tumor cells (≤0.2 mm) (41). Ultra-staging could upgrade 10–40% patients in previous studies for identification of low volume metastasis in lymph nodes (49, 50).

This meta-analysis showed no survival and recurrence detriment in SLN mapping compared with LND, in accordance with the previous meta-analysis (10). Experience from a study with 1,135 low risk patients indicated that 3-year OS and PFS were similar in two groups (P>0.07) (15). A multicenter study in high risk patients showed HR for association of staging approach (SLN and LND) with progression and death was 3.12 (95% CI 1.02–9.57) and 0.69 (95% CI 0.24–1.95) respectively (32). Similarly, Multinu et al (18). reported the risk of progression and death were not significantly different between SLN *vs.* LND (HR 1.27, 95% CI 0.6–2.67; HR 2.10, 95% CI 0.79–5.58, respectively). Additionally, no difference in recurrence pattern was observed between two groups; this meta-analysis showed there was no difference in overall recurrence (all sites), nodal recurrence, locoregional recurrence, and multifocal recurrence. Multiple studies reported distant/multifocal recurrence was predominant; Schiavone et al. (24) found 74% patients occurred multifocal recurrence and 16% endured nodal recurrence. A 56% (19/34) of multifocal recurrence in all patients with recurrence was reported in retrospective cohort study (18).

The imitations of the meta-analysis are as follows. First, most of pooled studies are retrospective cohort studies, futured prospective studies comparing SLN and LND are warranted. Second, some included studies did not provide the needed data directly, therefore some statistical methods were utilized to obtain proper data, which may decrease inaccuracy. Third, this meta-analysis is based on observational studies, and fails to reach high GRADE assessment.

In conclusion, the present meta-analysis reviewed current evidence on SLN mapping in comparison of LND. SLN is capable of reducing blood loss during operation in regardless of surgical approach with firm evidence from TSA. Future studies on operation time and complications are needed for further analysis. SLN mapping is more targeted for less node dissection and more detection of positive lymph nodes even in high risk patients with conclusive evidence from TSA. Utility of SLN yields no survival detriment in EC patients.

## Data Availability Statement

The original contributions presented in the study are included in the article/**Supplementary Material**. Further inquiries can be directed to the corresponding author.

## Author Contributions

Conceptualization: YG. Methodology: All authors. Project administration: YX. Supervision: YX. Writing—original draft: YG. Writing—review and editing: all authors. All authors contributed to the article and approved the submitted version.

## Funding

This work was supported by grants from the National Natural Science Foundation of China (No.81772783 and No. 81472446) and the Chinese Academy of Medical Sciences Initiative for Innovative Medicine (CAMS-2017-I2M-1-002) to YX.

## Conflict of Interest

The authors declare that the research was conducted in the absence of any commercial or financial relationships that could be construed as a potential conflict of interest.
