# Supplementary material for: Operative and Oncological Outcomes Comparing Sentinel Node Mapping and Systematic Lymphadenectomy in Endometrial Cancer Staging: Meta-Analysis With Trial Sequential Analysis
Source: Front Oncol. 2021 Jan 13;10:580128. doi: 10.3389/fonc.2020.580128 (PMC7838488; doi:10.3389/fonc.2020.580128)
Supplement: Supplementary file 1 [file DataSheet_1.docx]

Supplementary Material

### S1 Search strategy

**1 PubMed**

#1 "Endometrial Neoplasms"[Mesh] OR (cancer of corpus uteri) OR (uterine corpus cancer) OR (uterine cancer)

#2 (Sentinel node AND Lymphadenectomy)

#3 #1 AND #2

**2 EMBASE**

('endometrium cancer'/exp OR 'endometrium cancer') AND ('sentinel lymph node'/exp OR 'sentinel lymph node') AND ('lymphadenectomy'/exp OR 'lymphadenectomy')

**3 Cochrane library CENTRAL**

"endometrial cancer" in All Text AND "sentinel lymph node" in All Text AND "lymphadenectomy"

**4 Web of science**

TS=(((endometiral cancer) OR (cancer of corpus uteri) OR (uterine corpus cancer) OR (uterine cancer)) AND (sentinel node*) AND (lymphadenectomy))

**5 OVID**

1# (Endometrial cancer or cancer of corpus uteri or uterine corpus cancer or uterine cancer).af.

2# ((sentinel adj3 node$) and sentinel node$).af.

3# Lymphadenectomy.af.

4# 1 AND 2 AND 3

**6 Clinical trials.gov**

sentinel node AND lymphadenectomy AND Endometrial Cancer

**7 Controlled Trials meta Register (www.controlled-trials.com/mrct/search)**

Condition：endometrial cancer

**8 WHO (http://apps.who.int/trialsearch/AdvSearch.aspx)**

condition: Endometrial cancer

intervention: sentinel node
