# Supplementary material for: Operative and Oncological Outcomes Comparing Sentinel Node Mapping and Systematic Lymphadenectomy in Endometrial Cancer Staging: Meta-Analysis With Trial Sequential Analysis
Source: Front Oncol. 2021 Jan 13;10:580128. doi: 10.3389/fonc.2020.580128 (PMC7838488; doi:10.3389/fonc.2020.580128)
Supplement: Supplementary file 3 [file DataSheet_3.docx]

**Supplement figures 1-5**

Supplement figure 1

1A Operative time


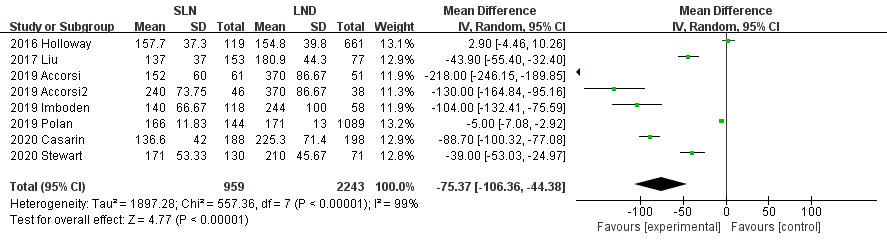


1B TSA of operative time


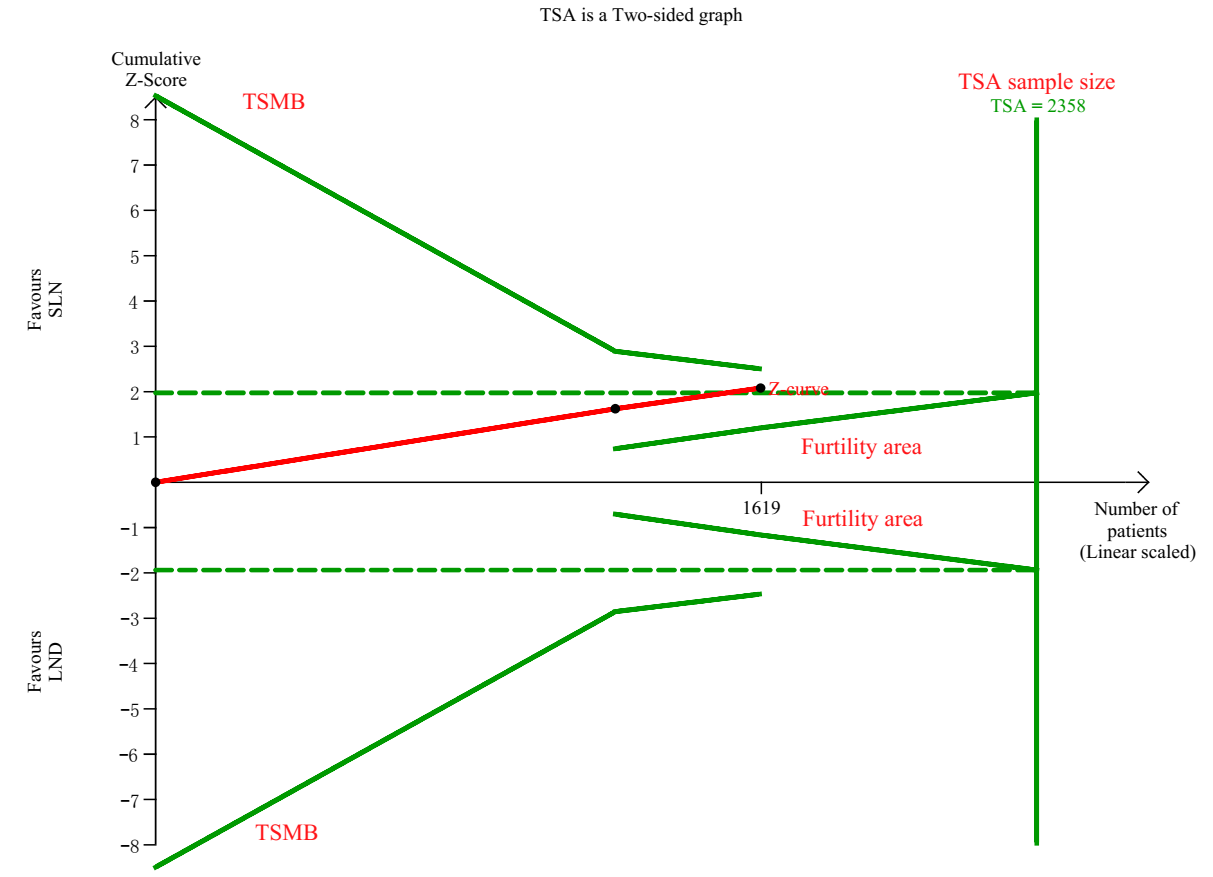


1C Frozen utility


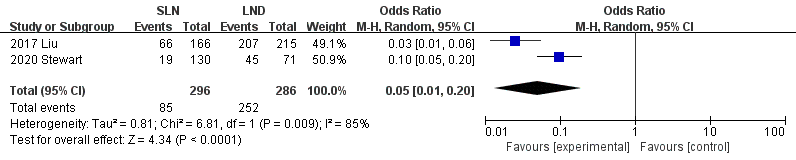


1D Length of stay


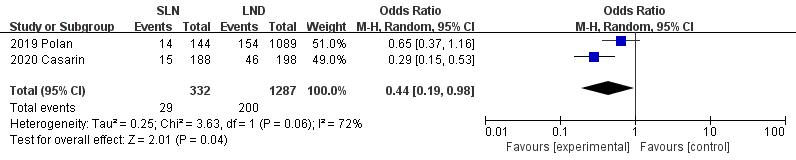


1E TSA of length of stay


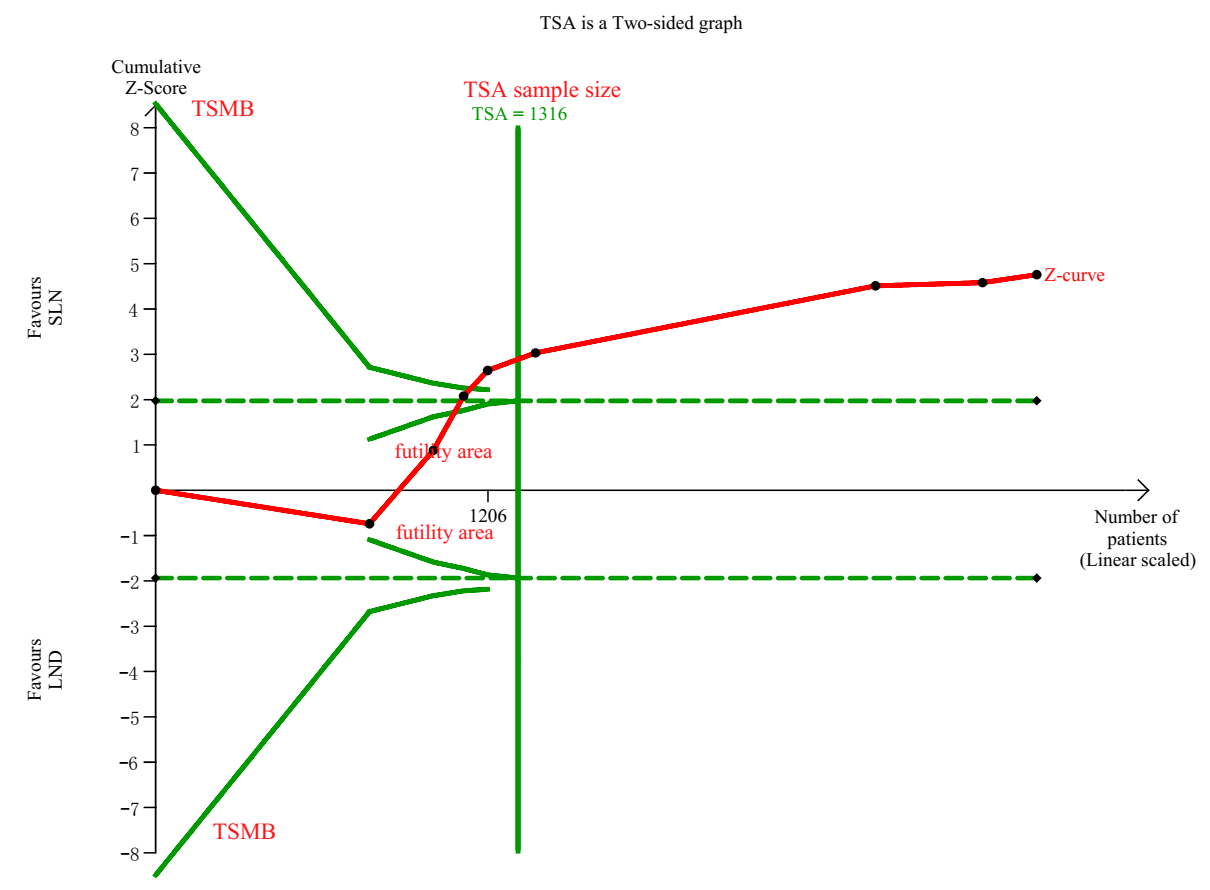


Supplement figure 1

(A) meta-analysis of operative time; (B) TSA of operative time, α=0.05, β=0.8, two-sided test; (C) meta-analysis of frozen utility; (D) meta-analysis of length of stay; (E) TSA of length of stay, α=0.05, β=0.8, two-sided test.

Supplement figure 2

2A P-LN+ in high risk patients


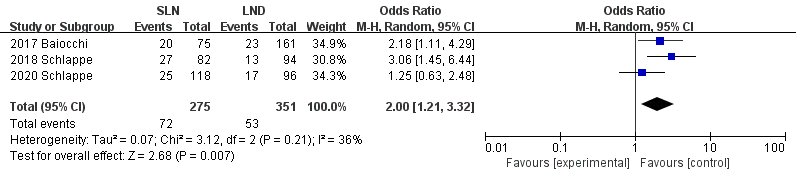


2B PA-LN+ in high risk patients


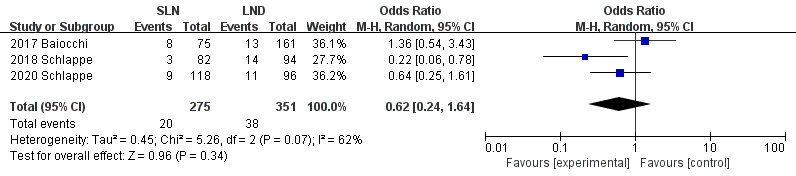


Supplement figure 2

(A) meta-analysis of pelvic lymph node positive (P-LN+) in high risk patients; (B) meta-analysis of para-aortic lymph node positive (PA-LN+) in high risk patients

Supplement figure 3

3A OS


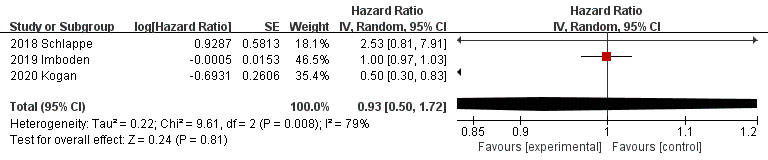


3B PFS


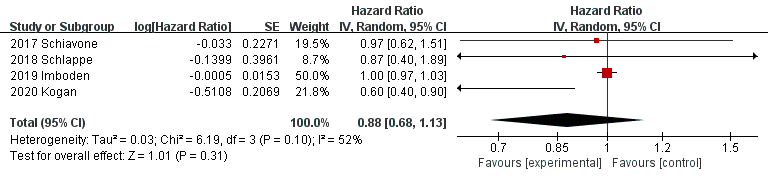


3C Overall recurrence


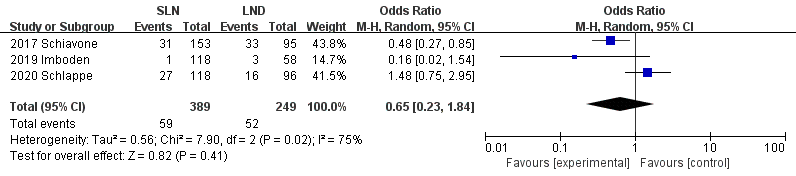


3D TSA of overall recurrence


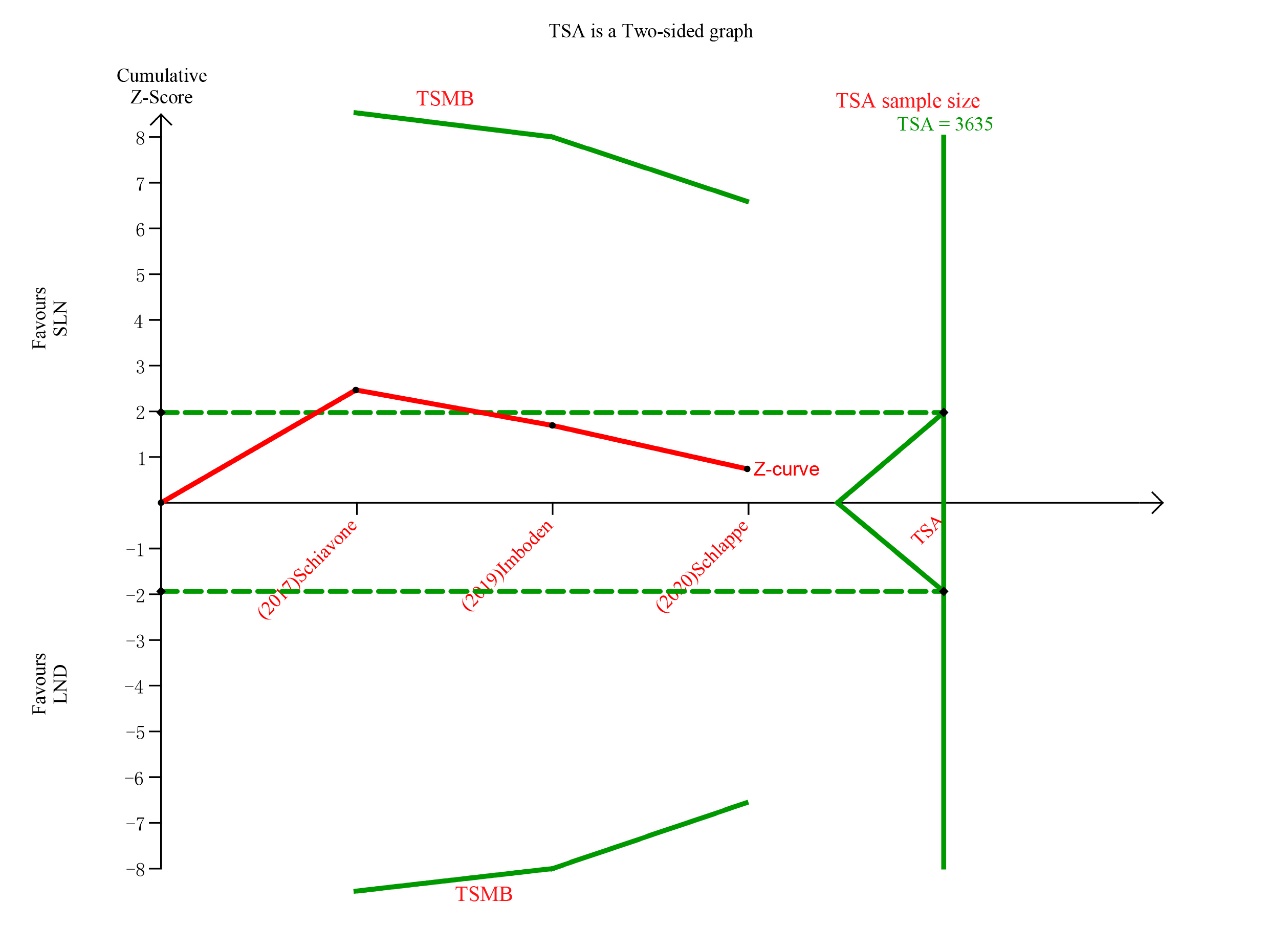


Supplement figure 3

(A) meta-analysis of overall survival (OS); (B) meta-analysis of progression-free survival (PFS); (C) meta-analysis of overall recurrence; (D) TSA of PA-LN removed, α=0.05, β=0.8, relative risk reduction=33.6%, incidence in control group=29.8%, two-sided test.

Supplement figure 4

4A nodal recurrence


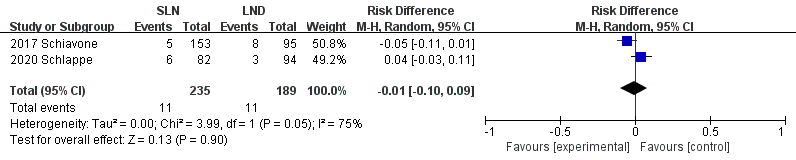


4B locoregional recurrence


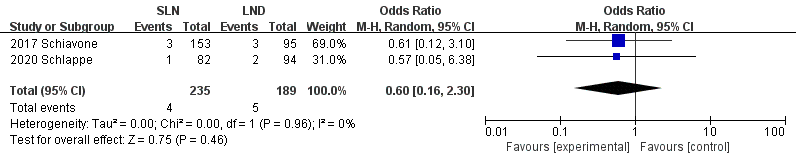


4C multifocal recurrence


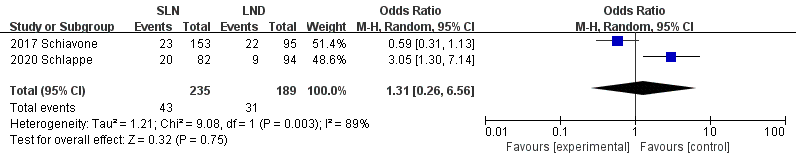


4D TSA of nodal recurrence


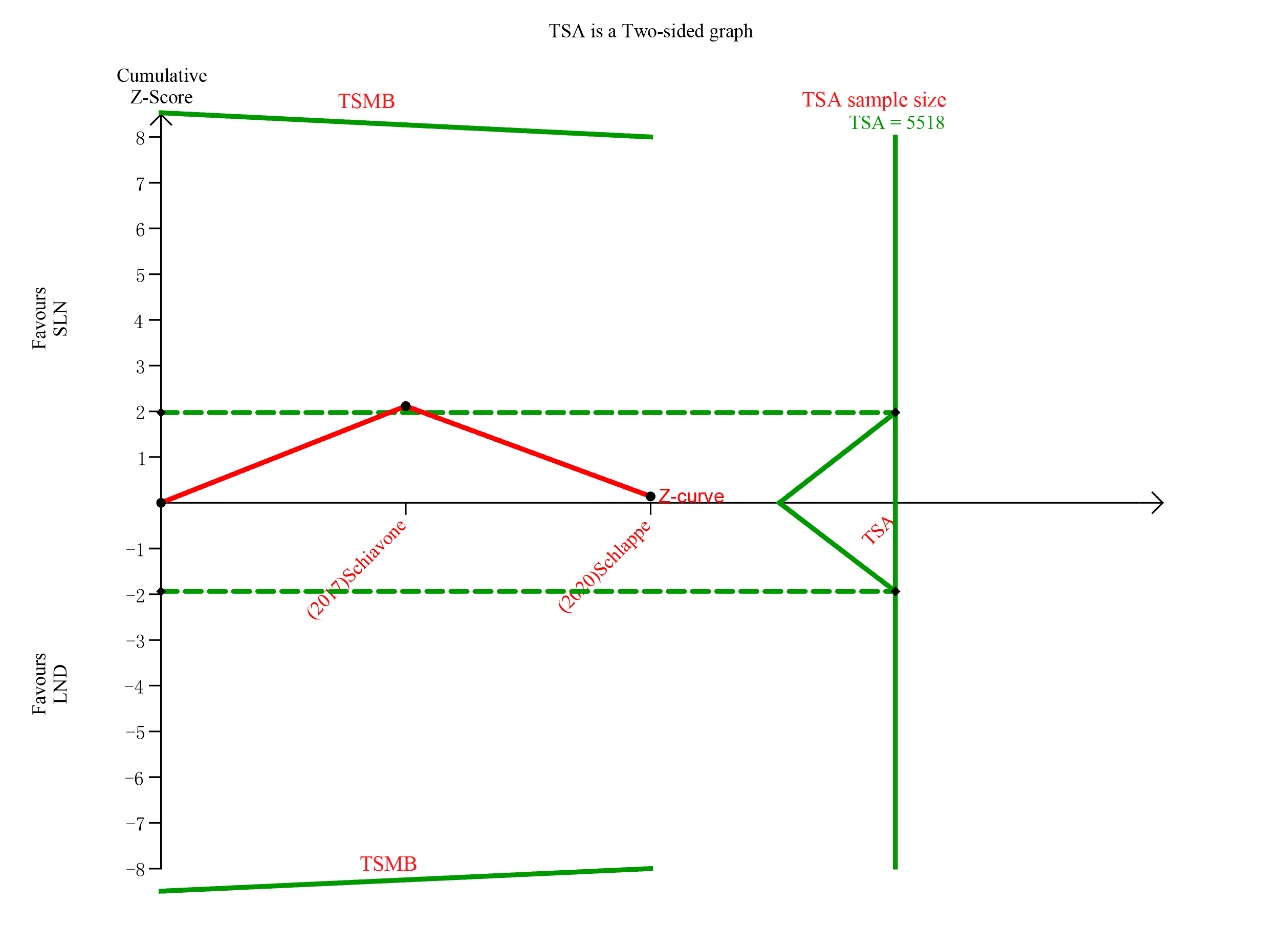


4E TSA of locoregional recurrence


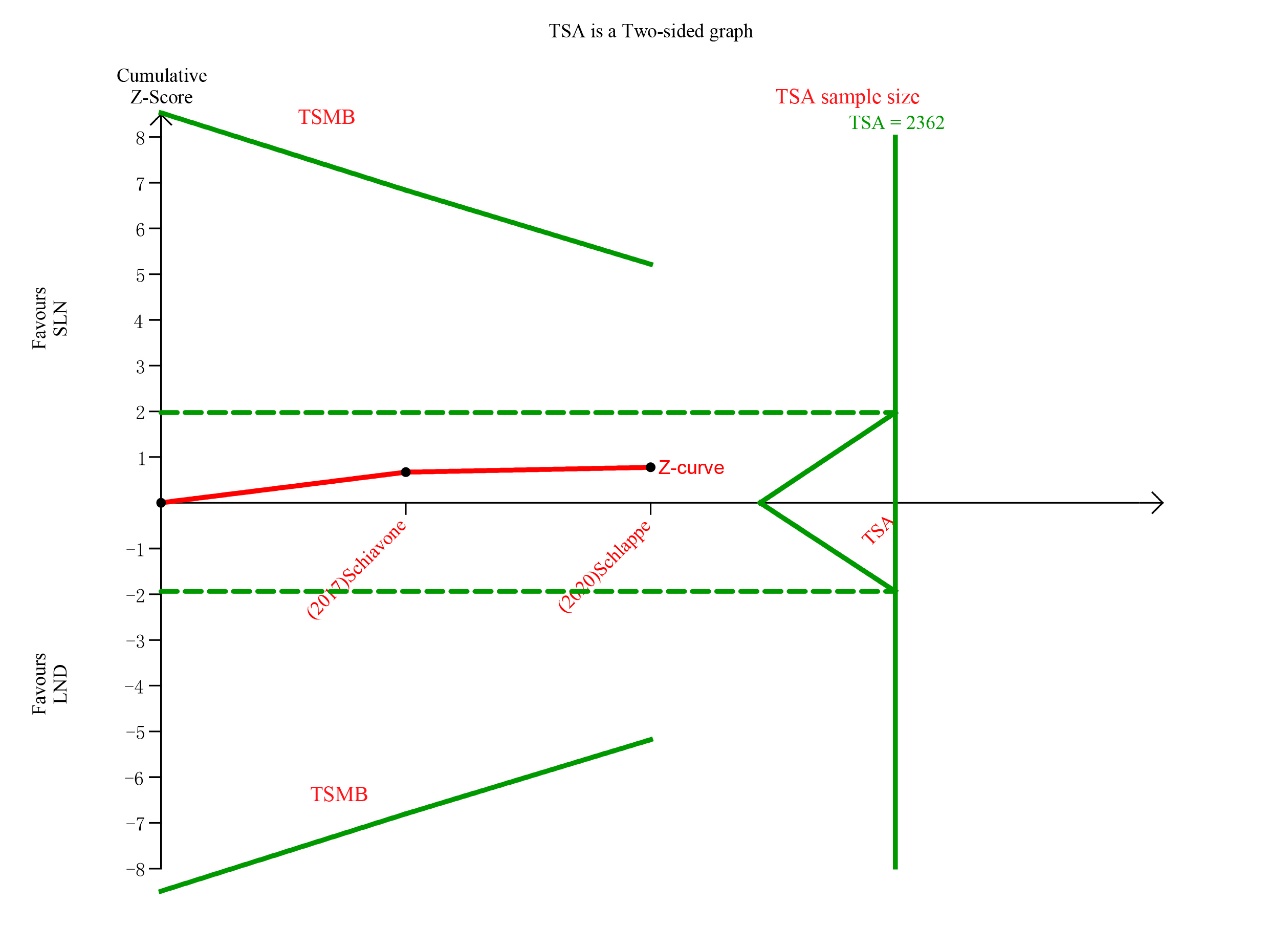


Supplement figure 4

(A) meta-analysis of nodal recurrence; (B) meta-analysis of locoregional recurrence; (C) meta-analysis of multifocal recurrence; (D) TSA of nodal recurrence, α=0.05, β=0.8, relative risk reduction=32.1%, incidence in control group=8.4%, two-sided test; (E) TSA of locoregional recurrence, relative risk reduction=52.9%, incidence in control group=3.4%, α=0.05, β=0.8, two-sided test.

Supplement figure 5

5 death of disease


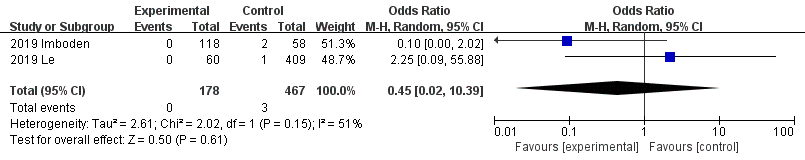


Supplement figure 5 meta-analysis of death of disease
