# Supplementary material for: Operative and Oncological Outcomes Comparing Sentinel Node Mapping and Systematic Lymphadenectomy in Endometrial Cancer Staging: Meta-Analysis With Trial Sequential Analysis
Source: Front Oncol. 2021 Jan 13;10:580128. doi: 10.3389/fonc.2020.580128 (PMC7838488; doi:10.3389/fonc.2020.580128)
Supplement: Supplementary file 5 [file Table_1.docx]

**Supplement tables 1-4**

Table S1.1 Characteristics of included studies in the meta-analysis

|  | Study design | institution | Study period | SLN | LND | Outcomes | NOS |
| --- | --- | --- | --- | --- | --- | --- | --- |
| 2020 Stewart | Retrospective cohort study | MD Anderson Cancer Center, USA | 2012; 2017 | 130 | 71 | Operative outcomes | 6 |
| 2020 Schlappe | Retrospective cohort study | Memorial Sloan Kettering Cancer Center, USA; Mayo Clinic, USA | 2006-2013; 2004-2008 | 118 | 96 | Nodal assessment  Oncological outcome | 7 |
| 2020 Casarin | Retrospective cohort study | Mayo Clinic, USA | 1/1/2009- 6/30/2016 | 188 | 198 | Operative outcomes | 7 |
| 2020 Bogani | Retrospective cohort study | Three Italian Gynecologic  Oncology Units | 01/01/2006 -12/31/2016 | 180 | 180 | Nodal assessment | 8 |
| 2019 Polan | Retrospective cohort study | Northwestern University, Chicago, USA | 1/1/1996-12/31/2017 | 144 | 1089 | Operative outcomes | 7 |
| 2020 Kogan | Retrospective cohort study | McGill University, Montreal, Quebec, Canada | Dec 2007 - Sept 2014 | 250 | 193 | Nodal assessment  Oncological outcome | 8 |
| 2019 Le | Retrospective cohort study | The Ottawa Hospital, Canada | 2004-2008; 2006-2013 | 60 | 409 | Nodal assessment  Oncological outcome | 8 |
| 2019 Imboden | Retrospective cohort study | University Hospital of Bern, Switzerland and Ospedale Santa Chiara di Trento, Italy | 2012-2016 | 118 | 58 | Operative outcomes Oncological outcome | 6 |
| 2019 Accorsi | Retrospective cohort study | Barretos Cancer Hospital, Brazil | 2004-2012 | 107 | 89 | Operative outcomes | 7 |
| 2018 Schlappe | Retrospective cohort study | Memorial Sloan Kettering Cancer Center, USA; Mayo Clinic, USA | April 2013-March 2018 | 82 | 94 | Nodal assessment Oncological outcome | 6 |
| 2017 Schiavone | Retrospective cohort study | MD Anderson cancer center, USA | 2005-2013; 2004-2008 | 153 | 95 | Nodal assessment  Oncological outcome | 8 |
| 2017 How | Retrospective and prospective cohort study | Jewish General Hospital, Canada | January 2005- July 2015 | 275 | 197 | Nodal assessment  Oncological outcome | 8 |
| 2017 Baiocchi | Retrospective cohort study | Brigham and Women's Hospital, USA | December 2007-June 2014 | 153 | 77 | Nodal assessment | 6 |
| 2017 Liu | Retrospective cohort study | AC camargo cancer center, Brazil | 1/1/2014-9/29/2016 | 75 | 161 | Operative outcomes  Nodal assessment | 7 |
| 2016 Holloway | Retrospective cohort study | Florida Hospital, USA | June, 2013- July, 2006 | 119 | 661 | Operative outcomes  Nodal assessment | 6 |

SLN: sentinel lymph node mapping; LND: lymphadenectomy; NOS: Newcastle Ottawa scale

Table S1.2 disease characteristics of included studies in the meta-analysis

|  | Age(SLN vs LND) | BMI(SLN vs LND) | FIGO stage | Grade | Histology | Risk stratification |
| --- | --- | --- | --- | --- | --- | --- |
| 2020 Stewart | 64.1 (27.6–87.1) vs 61.4 (30.7–84.5) | 33.9 (18.4–58.1) vs  33.9 (19.2–58.0) | I-IV | NR | Endometrioid and  non-endometrioid | High, median and low risk |
| 2020 Schlappe | 66.2 ±8.2 vs  66.3 ±11.6 | 29.8±6.6 vs  30.8±8.2 | I-III | G3 | serous and clear cell | High risk |
| 2020 Casarin | 63.9±10.8 vs  63.9±9.8 | 36.5±9.0vs  36.8±9.3 | NR | NR | NR | NR |
| 2020 Bogani | 60.88±11.0; 61.1± 10.1 vs 62.5±8.8 | 26.36±4.1; 27.3±6.7 vs 27.8±4.7 | I-III | G1-G3 | Endometrioid and  non-endometrioid | High, median and low risk |
| 2020 Kogan | 64(30–90) vs 63(39–92) | 30.7(16.9–63.3) vs  29.3(18.7–85.6) | I-IV | G1-G3 | Endometrioid and  non-endometrioid | High, median and low risk |
| 2019 Polan | 63.0 ±0.90 vs  64.4 ±0.31 | 36.5(30.3-40.8) vs  32.7 (27.4-39.0) | I-III | NR | NR | High, median and low risk |
| 2019 Le | 63.5 (36‐78) vs 62.5 (28‐86) | 32.8 (18‐60) vs  32.2 (21‐52) | I | G1-G3 | Endometrioid and  non-endometrioid | High, median and low risk |
| 2019 Imboden | 62.9 (32-92) vs 64.8 (38-86) | 28.0 (18-52) vs  29.9 (17-48) | I | G1-2 | NR | Low risk |
| 2019 Accorsi | 60(44-87);63(46-77) vs 62(31-80) | 33 (21.4-48.3); 29.3 (22.2-41.3) vs  30.4(18-46.3) | NR | G1-3 | Endometrioid, serous, clear cell, carcinosarcoma and other | High, median and low risk |
| 2018 Schlappe | 64.3 ±10.2 vs  69.2 ±10.92 | 29.8±6.8vs  31.6 ±8.1 | I-III | G1-3 | Endometrioid | High risk |
| 2017 Schiavone | 65 (45–89) vs  68 (45–85) | 29.6 (17.6–53.4) vs  29.3 (17.2–60.3) | I-IV | NR | Serous uterine carcinoma | High risk |
| 2017 How | 64±11.2 vs  65 ±11.4 | 31.6±8.8 vs  31.6±8.6 | I-III | G1-3 | Endometrioid, serous, clear cell, carcinosarcoma and other | High, median and low risk |
| 2017 Baiocchi | 64.5±10.7 vs  64.4± 10.4 | 31.7±8.2 vs  30.5±7.0 | I-IV | G1-3 | Endometrioid and  non-endometrioid | High risk |
| 2017 Liu | 61 (41–83) vs  61 (36–85) | 27.2 (17.9–43.7) vs  26.7 (16.2–58.3) | NR | G1-3 | Endometrioid, serous, clear cell, carcinosarcoma and other | High, median and low risk |
| 2016 Holloway | 65.5±9.2 vs  63.0±10.9 | 32.3±8.0 vs  31.7±7.6 | I-IV | G1-3 | Endometrioid and  non-endometrioid | High, median and low risk |

SLN: sentinel lymph node mapping; LND: lymphadenectomy; BMI: body mass index; NR: not recording

Table S2 Characteristics of included studies in operative outcomes

|  | SLN | LND | Approach | SLN procedure | Intra-op | Post-op | Op time (min) | Blood loss  (ml) | Conversation rate | Re-admission | Re-op | Length of stay(≥2d) | Frozen rate |
| --- | --- | --- | --- | --- | --- | --- | --- | --- | --- | --- | --- | --- | --- |
| 2020 Stewart | 130 | 71 | LA, robotic and Open | NR | 3vs1 | 4vs1 | 171 (96–416) vs 210 (92–366) | 75 (10–1500) vs 100 (20–2630) | 9vs4 | NR | NR | NR | 19 vs45 |
| 2020 Casarin | 188 | 198 | Robotic | Cervical injection of indocyanine green | 1vs4 | 1vs3 | 136.6±42.0vs 225.3±71.4 | 50 (50–100) vs 100 (60–200) | 1vs2 | 4vs7 | 0vs2 | 15vs46 | NR |
| 2019 Polan | 144 | 1089 | LA | NR | NR | 3vs39 | 166(138-209) vs171(133-211) | NR | NR | 2vs50 | 0vs6 | 14 vs154 | NR |
| 2019 Imboden | 118 | 58 | LA | Cervical injection of Verdye® | 0vs3 | 0vs3 | 140 (80-480) vs 244 (110-510) | 94 (10-400) vs 240 (50-1000) | NR | NR | NR | NR | NR |
| 2019 Accorsi | 107 | 89 | LA | Cervical injection of patent blue | 7vs10 | 7vs34 | 152(60-300) 240(125-420)  vs370(80-600) | 20(0-500) 45(0-500)  vs100(0-2300) | NR | NR | NR | NR | NR |
| 2017  Liu | 75 | 161 | LA | Cervical injection of indocyanine | NR | 8vs10 | 137.0±37.0 vs 180.9±44.3 | 56.2±56.7 vs 80.1±61.4 | NR | NR | NR | NR | 66vs207 |
| 2016 Holloway | 119 | 661 | Robotic | Cervical injection of indocyanine | NR | NR | 157.7(±37.3) vs 154.8 (±39.8) | NR | NR | NR | NR | NR | NR |

SLN: sentinel lymph node mapping; LND: lymphadenectomy; NR: not recording; LA: laparoscopy; Op: operation; NR: not recording

Table S3 Characteristics of included studies in nodal assessment

|  | SLN group | LND group | SLN intervention | LND intervention | Macro-metastasis | Micro-metastasis | Isolated tumor cells | |
| --- | --- | --- | --- | --- | --- | --- | --- | --- |
| 2020 Schlappe | 118 | 96 | SLN±P-LND±PA-LND | P-LND±PA-LND | NR | NR | | NR |
| 2020 Bogani | 180 | 180 | SLN±P-LND±PA-LND | P-LND±PA-LND | 10vs13 | 5vs0 | | 11vs0 |
| 2020 Kogan | 250 | 193 | SLN+P-LND±PA-LND | P-LND+PA-LND | NR | NR | | NR |
| 2019 Le | 60 | 409 | SLN±P-LND±PA-LND | P-LND±PA-LND | NR | NR | | NR |
| 2018 Schlappe | 82 | 94 | SLN±P-LND±PA-LND | P-LND±PA-LND | NR | NR | | NR |
| 2017 Schiavone | 153 | 95 | SLN±P-LND±PA-LND | P-LND±PA-LND | NR | NR | | NR |
| 2017 How | 275 | 197 | SLN±P-LND±PA-LND | P-LND±PA-LND | NR | NR | | NR |
| 2017 Baiocchi | 153 | 77 | SLN+P-LND±PA-LND | P-LND±PA-LND | NR | NR | | NR |
| 2017 Liu | 75 | 161 | SLN±P-LND±PA-LND | P-LND±PA-LND | NR | NR | | NR |
| 2016 Holloway | 119 | 661 | SLN+P-LND±PA-LND | P-LND±PA-LND | 14vs74 | 10vs17 | | 12vs6 |

SLN: sentinel lymph node mapping; LND: lymphadenectomy; P-LND: pelvic lymphadenectomy; PA-LND: para-aortic lymphadenectomy; NR: not recording

Table S4 Characteristics of included studies in oncologic outcomes

|  | SLN group | LND group | Mean follow up time | Risk | Peritoneal cytology positive | LVSI+ | MI≥50% | Cervical  Stroma + | Adjuvant therapy | OS | PFS | Recurrence | Death of disease |
| --- | --- | --- | --- | --- | --- | --- | --- | --- | --- | --- | --- | --- | --- |
| 2020 Schlappe | 118 | 96 | 2.3y vs 3.2y | Mixed | 19vs16 | 44vs25 | 28vs23 | 11vs4 | 84%vs40% | 3-year 88% vs 77% | NR | 27vs16 | 29 |
| 2020 Kogan | 250 | 193 | 6.3y vs 8.9y | Mixed | NR | 55vs55 | NR | NR | 29.2% vs 26.4% | 6-year 90.0% vs 81.0% | 6-year 85.0% vs 75.0% | NR | NR |
| 2019 Le | 60 | 409 | 10.6m | Mixed | NR | NR | NR | NR | RT28.3%vs34%CT12%vs6.8% | NR | NR | NR | 0vs1 |
| 2019 Imboden | 118 | 58 | 33 m | Low | NR | 3vs10 | NR | NR | 16.7% | 97.75%vs 93.59% | 96.84%vs76.91% | 1vs3 | 0vs2 |
| 2018 Schlappe | 82 | 94 | 2.6y vs 2.8y | High | 12vs18 | 61vs29 | all | 11vs15 | 90.2%vs70.2% | 3-year 91.8% vs 77.6% | 3year78.7%vs  77.7% | NR | NR |
| 2017 Schiavone | 153 | 95 | 31.5m vs 76.8m | Mixed | NR | 55vs46 | 34vs28 | NR | 80%vs83% | NR | 2-year 77% vs 71% | 31vs33 | NR |
| 2017 How | 275 | 197 | 33m vs 40 m | Mixed | NR | 61vs69 | NR | NR | RT62.2%vs53.8%;CT31.3vs26.9% | NR | NR | 26vs28 | NR |

SLN: sentinel lymph node mapping; LND: lymphadenectomy; LVSI: lymph vascular space invasion; MI: myometrial invasion; RT: radiotherapy; CT: chemotherapy; NR: not recording
